# Estimated crop yield losses due to surface ozone exposure and economic damage in India

**DOI:** 10.1007/s11356-014-2657-6

**Published:** 2014-02-28

**Authors:** S. B. Debaje

**Affiliations:** Indian Institute of Tropical Meteorology, Dr. Homi Bhabha Road, Pashan Pune, 411008 India

**Keywords:** Air pollution impacts, Surface ozone, Crop yield losses, Economical losses, Food security, Atmospheric condition

## Abstract

In this study, we estimate yield losses and economic damage of two major crops (winter wheat and rabi rice) due to surface ozone (O_3_) exposure using hourly O_3_ concentrations for the period 2002–2007 in India. This study estimates crop yield losses according to two indices of O_3_ exposure: 7-h seasonal daytime (0900–1600 hours) mean measured O_3_ concentration (M7) and AOT40 (accumulation exposure of O_3_ concentration over a threshold of 40 parts per billion by volume during daylight hours (0700–1800 hours), established by field studies. Our results indicate that relative yield loss from 5 to 11 % (6–30 %) for winter wheat and 3–6 % (9–16 %) for rabi rice using M7 (AOT40) index of the mean total winter wheat 81 million metric tons (Mt) and rabi rice 12 Mt production per year for the period 2002–2007. The estimated mean crop production loss (CPL) for winter wheat are from 9 to 29 Mt, account for economic cost loss was from 1,222 to 4,091 million US$ annually. Similarly, the mean CPL for rabi rice are from 0.64 to 2.1 Mt, worth 86–276 million US$. Our calculated winter wheat and rabi rice losses agree well with previous results, providing the further evidence that large crop yield losses occurring in India due to current O_3_ concentration and further elevated O_3_ concentration in future may pose threat to food security.

## Introduction

Surface ozone (O_3_) is a secondary air pollutant, despite its detrimental effects on human health, field experiments has demonstrated that the O_3_ causes substantial damage to plant and agricultural crops (Amann et al. 2008; Fuhrer 2009). Exposure of high O_3_ concentration greater than 40 parts per billion by volume (ppbv) causes large yield losses of many agriculture crops (Fuhrer and Ashmore 1997). Over 90 % of vegetation is damaged due to O_3_ exposure alone (Felzer et al. 2007). Several field experiments studies in the USA, Europe, and Asia have demonstrated that the O_3_ is responsible for more damage to vegetation and agricultural crops than any other air pollutant (Mauzerall and Wang 2001; Mittal et al. 2007; Engardt 2008; Ghude et al. 2008; The Royal Society 2008; Booker et al. 2009; Emberson et al. 2009; Zhu et al. 2011; Wilkinson et al. 2012; Tang et al. 2013; Elampari et al. 2013 and references therein). Field experiment studies in the USA (National Crop Loss Assessment Network, NCLAN) and in Europe (European Open Top Chambers Programme, EOTCP) showed that crops yield losses occur by 5–10 % and deteriorating a crop quality and larger losses are expected in the future. Relatively less number of experimental studies has been conducted in Asia and in developing countries compared to in the USA and in Europe. However, a few experimental and modeling studies have been performed demonstrating that crops yield losses occurring due to O_3_ exposure in Asia and in particular in India and Pakistan (Agrawal 2003; Wahid 2003; Wang and Mauzerall 2004). The winter wheat losses estimated greater than 10 % due to high O_3_ levels (>60 ppbv) in southern China using an atmospheric chemistry model along with a regional climate model (Chameides et al. 1999). In the Indian subcontinent, studies performed by Agrawal (2003), Wahid (2003), and Debaje et al. (2010) documented that winter wheat yield losses by 5–43 % due to O_3_ exposure in winter season. The O_3_-induced agriculture losses are two to three times larger than estimated crop losses due to climate change that highlights the development of crop cultivars with O_3_-resistance are beneficial in future (Lobell and Field 2007).

Global studies relating to O_3_-induced crop yield losses has shown that relative yield loss (RYL), crop production loss (CPL), and associated economic cost losses (ECL) highest in India followed by in China (Van Dingenen et al. 2009; Avnery et al. 2011a). Van Dingenen et al. (2009) reported that highest wheat and rice losses in India due to O_3_ damage using M7 and AOT40 indices. Similarly, Avnery et al. (2011a) reported that highest wheat losses in India from M7 and AOT40 indices due to O_3_ damage. To our knowledge, only the two studies are reported O_3_-induced crop yield losses on a global scale, highlighting that the highest winter wheat and rice losses in India. China, Japan, and South Korea are also experiencing similar wheat and rice losses on large scale in Asian region by O_3_ damage due to increase of O_3_ concentration (Wang and Mauzerall 2004; Wang et al. 2012).

We use M7 and AOT40 indices to estimate winter wheat and rabi rice crop yield losses using O_3_ data available in India. The M7 index is the seasonal daylight mean O_3_ concentration for the 7 h mean (09–16 hours) and AOT40 (accumulation exposure of O_3_ concentration over a threshold of 40 ppbv during daylight hours, 07–18 hours) index for 3-month period from growing to harvesting season of crop. In India, winter wheat is mainly cultivated from December to March during the winter season. Further, rice is grown in two major seasons, kharif (June–October) and rabi (October–February) (NCS 2013). Most of the rice area (39.3 million ha) and production are in the kharif season (86 % of the total rice production). However, the productivity of kharif rice is lower 2,985 kg ha^−1^ than the rabi rice productivity 4,691 kg ha^−1^. The highest area under rabi rice cultivation is 1.5 and 1.4 million ha in Andhra Pradesh and in West Bengal, respectively, of the total area 4.3 million ha.

The photochemical production of O_3_ is controlled by oxides of nitrogen (NO_x_) in India implies that O_3_ concentration increases with increase of NO_x_ concentration (Berntsen et al. 1996; van Aardenne et al. 1999; Lelieveld et al. 2001). The daytime maximum of O_3_ concentrations is less than 40 ppbv during the kharif rice season help sparing from O_3_ damage because of south west (SW) monsoon rains (June–September; IMD 2007; http://www.imd.gov.in). Rice is moderately sensitive to O_3_; its AOT40 critical limit is 12.8 ppm h, while winter wheat is sensitive to O_3_; its AOT40 critical limit is 3.3 ppm h for 5 % yield losses (Mill et al. 2007). In this study, therefore, our analysis is on rabi rice losses when daytime maximum of O_3_ concentration greater than 40 ppbv. The production of kharif crops (particularly kharif rice) totally depends on the SW monsoon rains which are highly erratic in nature (DRD 2007). In this study, we present mean RYL, CPL, and associated ECL are occurred for two major staple crops winter wheat and rabi rice due to O_3_ pollution for five crop years for the period 2002–2007 (study period) using O_3_ exposure yield response indices M7 and AOT40 calculated first time from measured hourly O_3_ concentrations available data over the Indian region.

## Materials and methods

We estimate winter wheat and rabi rice yield losses using O_3_ exposure indices M7 and AOT40 for the study period, except for the period 2008–2009 (Singla et al. 2011) and 2009–2011 (Mahapatra et al. 2012) as O_3_ studies are not available at these sites from 2002 to 2007 (Table 1). M7 index is calculated for all the available sites using monthly diurnal variation of O_3_ for 7 h mean from 0900 to 1600 hours daylight for a 3-month period from sowing to harvesting “growing season” of the crop. Winter wheat is grown between the months mid-November to April, while rabi rice grown from November–February to March–June. Average M7 index values vary between 49.5 and 68 ppbv over the Indian region. The reference concentration of O_3_ is 25 ppbv in the calculation of M7 index. AOT40 index is calculated from M7 index values obtained from measured O_3_ concentration and the relationship between 3-month AOT40 (parts per million hours, ppm h) and 3-month 7-h mean O_3_ (ppbv) provided by Mills et al. (2007; Table 2).

**Table 1:** Winter wheat and rabi rice crop yield losses are based on M7 and AOT40 indices of hourly surface ozone (O_3_) concentration (parts per billion by volume) during daylight hours measurements available data after the year 2000 in India

Site/State	Latitude Longitude	Altitude (m)	Period	O_3_ (ppbv)^a^	Reference
Northern India (21–35°N, 68–90°E)					
Nainital, UC	29.4°N 79.5°E	1958	2006–2008	42 ± 6 to 57 ± 11	Kumar et al. (2010)
Delhi, Delhi	28.4°N 77.5°E	216	1997–2004	50 ± 15 to 95 ± 22	Jain et al. (2005)
Agra, UP	27.2°N 78°E	169	2008–2009	55 ± 7 to 65 ± 11	Singla et al. (2011)
Agra, UP	27.2°N 78°E	169	2000–2002	56 ± 3 to 63 ± 5	Satsangi et al. (2004)
Kolkata, WB	22°N 88°E	5	2003–2004	25 ± 5 to 35 ± 7	Purkait et al. (2009)
Bhubaneswar, OR	21.3°N 85.3°E	45	2009–2011	50 ± 15 to 78 ± 23	Mahapatra et al. (2012)
Southern India (5-20°N, 68–90°E)					
Joharapur, MH	19.3°N 75.2°E	474	2002–2005	53 ± 17 to 55 ± 11	Debaje and Kakade (2009)
Ahmednagar, MH	19.1°N 74.8°E	657	2006–2007	42 ± 12 to 57 ± 15	Debaje et al. (2010)
Pune, MH	18.5°N 73.8°E	559	2003–2004	34 ± 20 to 55 ± 25	Beig et al. (2007)
Pune, MH	18.5°N 73.8°E	559	2001–2005	48 ± 7 to 50 ± 6	Debaje and Kakade (2009)
Anantapur, AP	14.6°N 77.6°E	331	2002–2003	47 ± 6 to 49 ± 8	Reddy et al. (2008)
Anantapur, AP	14.6°N 77.6°E	331	2001–2003	48 ± 6 to 56 ± 8	Ahammed et al. (2006)
Trivandrum, KR	8.6°N 77°E	2	2007–2009	25 ± 5 to 45 ± 10	David and Nair (2011)

*UC* Uttaranchal, *UP* Uttar Pradesh, *WB* West Bengal, *OR* Orissa, *MH* Maharashtra, *AP* Andhra Pradesh, *KR* Kerala

^a^Monthly average O_3_ concentration from December to March

**Table 2:** Air quality indices used to evaluate the winter wheat and rabi rice crop yield losses over India. All surface ozone (O_3_) concentration (parts per billion by volume) refers to hourly values

Index	Unit	Definition	Exposure/dose response function: relative yield loss (RYL)	Wheat		Rice		Reference
				a	b	a	b	
M7	ppbv	7-h seasonal O_3_ mean 3 months, 09–16 h	1 − exp[−(M7/a)^b^]/exp[−(25/a)^b^]	137	2.34	202	2.47	Wang and Mauzerall (2004)
AOT40	ppm h	(M7-29)/1.59	aAOT40	0.0161	0.99	0.00415	0.94	Mills et al. (2007)
		3 months						Van Dingenen et al. (2009)

AOT40 = ∑ ^*n*^_*i* = 1_[O_3_]_*i*_ − 40, [O_3_] > 40 ppbv; during daylight (0700–1800 hours; [O_3_] = hourly averaged O_3_ concentration in parts per billion by volume; 40 = threshold limit of O_3_ and *n* is the number of hours from growing to harvesting season of crop

*3 months* January, February, and March for wheat; December, January, and February for rice

Seasonal O_3_ mean of M7 and AOT40 indices are derived for winter wheat from January to March, and for rabi rice from December to February. Potential grain yield is primarily determined before heading, but actual yield, which is based on the amount of starch that ultimately fills the spikelet’s, is largely determined after heading (NCS 2013). Hence, we calculate O_3_ exposure yield response M7 index for last 3 months of the winter wheat (January–March) and rabi rice (December–February) crop. We select the top ten states which are major producers of winter wheat and rabi rice for assessing impact of O_3_ exposure according to the M7 and AOT40 indices (http://dwd.dacnet.nic.in/wheat_prod1/index.htm and http://drd.dacnet.nic.in/Downloads/Handbook-of-Statistics-2007.pdf) (DRD 2007).

## O_3_ exposure and CR relationships

The O_3_ indices are derived from open-top chamber (OTC) field studies conducted in the USA and Europe have established crop-specific crop response (CR) functions that used to predict the yield response of a crop to a given concentration of O_3_ exposure. We use M7 and AOT40 exposure-based indices and its CR relationships to calculate RYL of winter wheat and rabi rice shown in Table 2.

Winter wheat and rabi rice RYL is translated into CPL using mean production of winter wheat and rabi rice:

1 $$ \mathrm{CPL}=\frac{\mathrm{RYL}}{1-\mathrm{RYL}}\times {\mathrm{production}}_{2002-2007} $$

Winter wheat and rabi rice CPL is converted into ECL based on the mean minimum support price (MSP) for the five crops years (CACP 2012). The MSP for wheat and rice is 142.05 and 134 US$ per ton, respectively (1 t = 1,000 kg, 1 US$ = 44.49 Indian Rupees) fixed by the Government of India each year (http://agricoop.nic.in/Agristatistics.htm, http://cacp.dacnet.nic.in/Rpp/kharif_Report_2012-13.pdf). The ECL is calculated as follows:

2 $$ \mathrm{ECL}=\mathrm{CPL}\times {\mathrm{MSP}}_{2002-2007} $$

MSP is approximately 50 % less than the market price.

## Results

### RYL of winter wheat and rabi rice

**Fig. 1:**
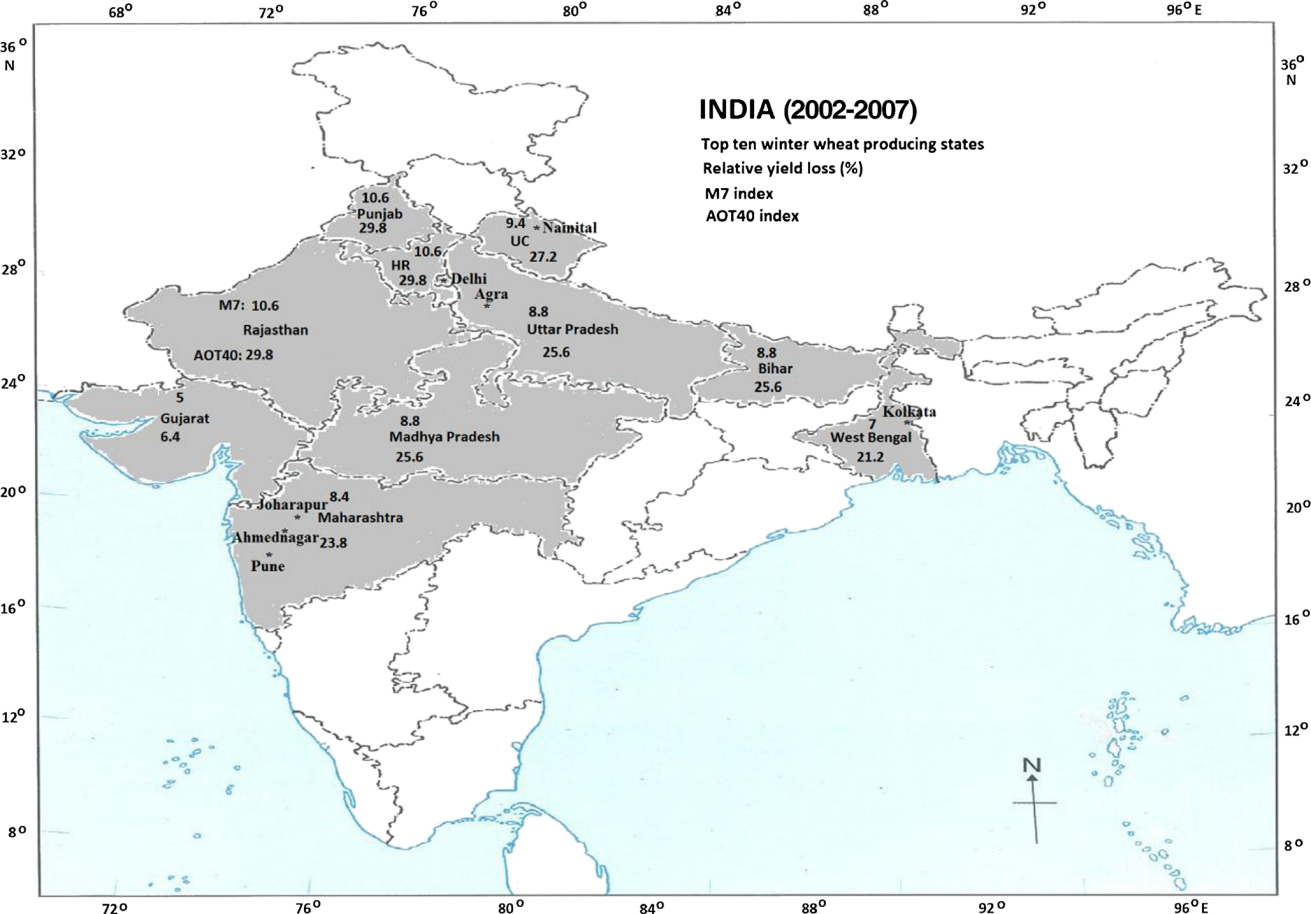
Map of India shows the winter wheat relative yield loss (in percent) per year in top ten states producing winter wheat for the period 2002–2007 due to O_3_ exposure according to the M7 and AOT40 indices. HR is Haryana state. Top numbers on the states name is RYL using M7 index and bottom number RYL using AOT40 index. *Stars* the location of O_3_ measurements site

Figure 1 illustrates the winter wheat RYL (in percent) in the top ten states (Uttar Pradesh, Punjab, Haryana, Madhya Pradesh, Rajasthan, Bihar, Maharashtra, Gujarat, West Bengal, and Uttaranchal) in India’s map due to O_3_ exposure according to the M7 and AOT40 indices per year for the study period (shaded areas). The production of winter wheat is low in the remaining states (unshaded areas). The winter wheat RYL is ranged between 5 and 11 % (6 and 30 %) using M7 (AOT40) index. The highest winter wheat RYL from 9 to 11 % (26–30 %) using M7 (AOT40) index is in Punjab, Haryana, Rajasthan, Uttaranchal, Uttar Pradesh, Bihar, and Madhya Pradesh in the Indo-Gangetic plain of India which is the most agriculture fertile region. All these states in northern India are experiencing high O_3_ concentration (>80 ppbv) during winter and summer season due to high emission of NO_x_ and favorable atmospheric conditions for photochemical O_3_ formation (van Aardenne et al. 1999; Ohara et al. 2007). The low winter wheat RYL 5–8 % (6–24 %) using M7 (AOT40) index was in Maharashtra, West Bengal, and Gujarat due to low O_3_ concentration. *We calculate the average winter wheat RYL is 9* % (*21* %) *using the M7* (*AOT40*) *index for the Indian region*. Winter wheat crop is especially sensitive to O_3_ in India due to the likely co-occurrence of peak levels of O_3_ and growing season (Emberson et al. 2003; Jain et al. 2005). The latitudinal variations of O_3_ concentration over the India show the higher O_3_ in northern India than in southern India (Carmichael et al. 2003; Table 1).

**Fig. 2:**
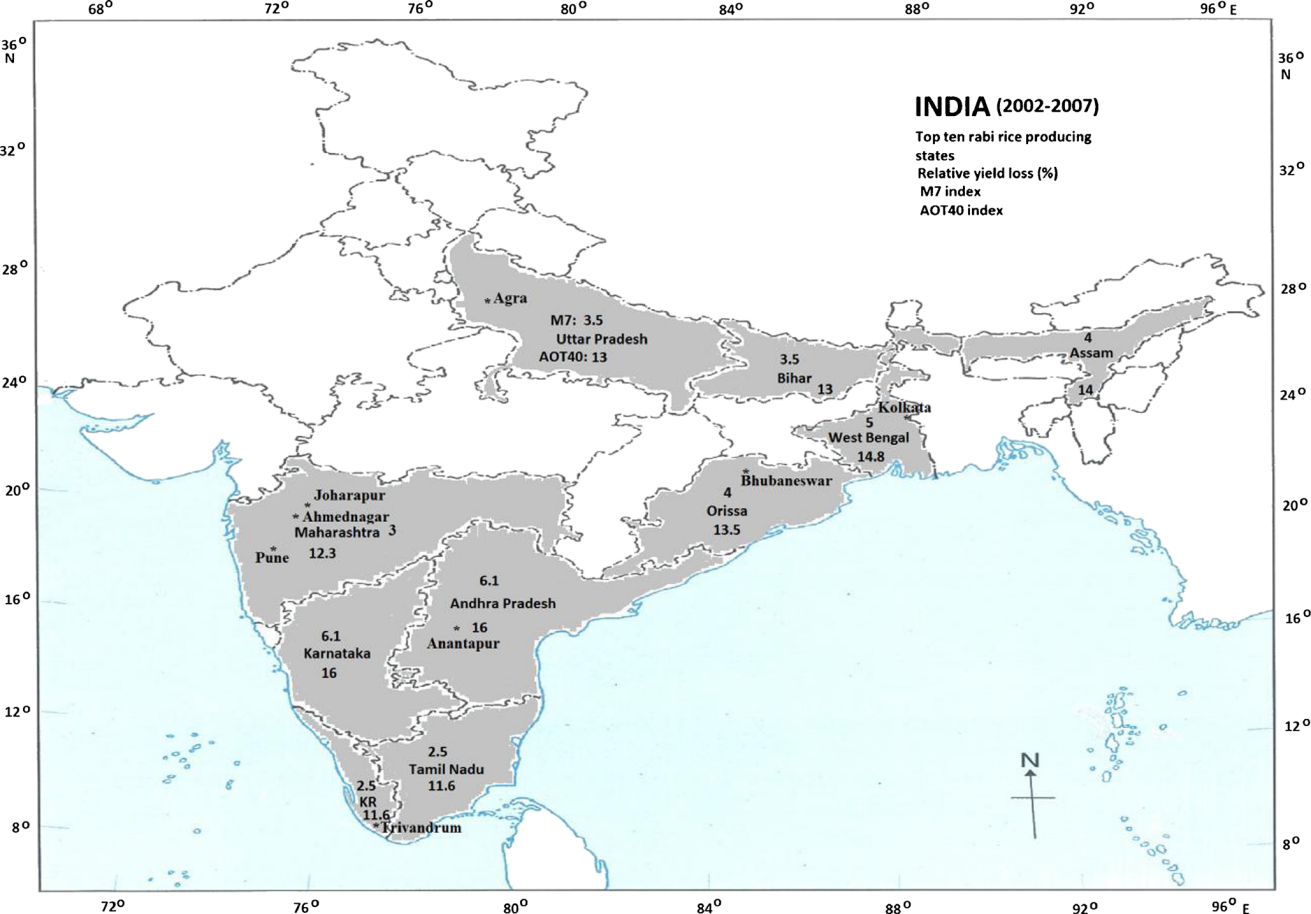
Map of India shows the rabi rice relative yield loss (in percent) per year in top ten states producing rabi rice for the period 2002–2007 due to O_3_ exposure according to the M7 and AOT40 indices. Star mark shows the location of O_3_ measurements site

Similarly, Fig. 2 illustrates the rabi rice RYL in the top ten states (West Bengal, Andhra Pradesh, Karnataka, Orissa, Assam, Tamil Nadu, Bihar, Kerala, Maharashtra, and Uttar Pradesh) due to O_3_ exposure according to the M7 and AOT40 indices (shaded areas). The production of rabi rice is low in the remaining states (unshaded areas). The rabi rice RYL ranged between 3 and 6 % (12 and 16 %) using the M7 (AOT40) index. The highest rabi rice RYL is 6 % (16 %) using M7 (AOT40) index in Andhra Pradesh and Karnataka in the southern India, while the lowest rabi rice RYL is 3 % (12 %) in Kerala and Tamil Nadu in the southern tip of India. The average rabi rice RYL is 5 % (14 %) using M7 (AOT40) index for the Indian region.

### Winter wheat losses and associated monetary loss

Table 3 presents the mean winter wheat production and winter wheat losses (RYL, CPL, and ECL) estimated in the top ten states per year using M7 and AOT40 indices for the study period. The production of winter wheat in other states in India is low and it is shown as others (16 states) in Table 3. *The highest winter wheat production is 27 million metric tons* (*Mt*) *occurred in Uttar Pradesh followed by in Punjab 17 Mt and Haryana 10 Mt account for nearly 70* % *of the total winter wheat production is 81 Mt per year*. However, the highest yield of winter wheat is 5,513 kg/ha in Punjab, 4,947 kg/ha in Haryana, and 3,275 kg/ha in Uttar Pradesh. The winter wheat production was only 10 Mt (12 %) from Bihar, Maharashtra, Gujarat, West Bengal, and Uttaranchal of the total winter wheat production because of low yield (1,594–2,791 kg/ha). Table 3 also shows the winter wheat losses estimated using M7 and AOT40 indices. The higher winter wheat RYL is 5 Mt (15 Mt) in top three states of the total winter wheat RYL 8 Mt (22 Mt) per year than in the remaining states using M7 (AOT40) index. *The mean total winter wheat RYL is 8 Mt* (*22 Mt*) *account for 10* % (*27* %) *losses using M7* (*AOT40*) *index of the total winter wheat productions in these three states*. Winter wheat CPL is calculated using winter wheat RYL and winter wheat production by Eq. (1). *The highest winter wheat CPL 3 Mt* (*9 Mt*) *occurred in Uttar Pradesh followed by 2 Mt* (*7 Mt*) *and 1 Mt* (*4 Mt*) *in Punjab and Haryana*, *respectively, of the total winter wheat CPL 9 Mt* (*29 Mt*) *per year using M7* (*AOT40*) *index. Using the AOT40 index*, *winter wheat CPL is 20 Mt* (*70* %) *in top three states of the total winter wheat CPL 29 Mt*. The highest winter wheat CPL is 17 Mt (∼60 %) in Utter Pradesh and Punjab together of the total winter wheat CPL. The associated winter wheat ECL is calculated using winter wheat CPL and winter wheat MSP by Eq. (2). The highest winter wheat ECL is 841 million US$ (2,885 million US$) of the total winter wheat ECL 1,222 million US$ (4,091 million US$) using M7 (AOT40) index in the top three states annually.

**Table 3:** Mean winter wheat production (WP) and winter wheat losses (relative yield loss (RYL), crop production loss (CPL) and economic cost loss (ECL)) per year in top ten states in India using M7 and AOT40 indices for the period 2002–2007

State	Winter wheat production	WP (%)	Winter wheat losses					
			M7			AOT40		
			RYL	CPL	ECL	RYL	CPL	ECL
Uttar Pradesh	27.36	33.7	2.41	2.64	375	7	9.41	1,336.7
Punjab	17.24	21.2	1.83	2.04	289.8	5.14	7.3	1,037
Haryana	10.47	12.9	1.11	1.24	176.1	3.12	3.6	511.4
Madhya Pradesh	7.79	9.6	0.69	0.75	106.5	1.99	2.68	380.7
Rajasthan	7.2	8.9	0.76	0.85	120.7	2.14	3.06	434.7
Bihar	5.02	6.2	0.44	0.48	68.2	1.29	1.73	245.7
Maharashtra	1.39	1.7	0.12	0.13	18.5	0.33	0.43	61.1
Gujarat	1.27	1.6	0.06	0.07	9.9	0.08	0.09	12.8
West Bengal	1.009	1.2	0.07	0.08	11.4	0.21	0.27	38.3
Uttaranchal	0.84	1	0.08	0.09	12.8	0.23	0.31	44
Others (16 states)	1.7	2	0.17	0.19	27	0.25	0.3	42.6
Total (all India)	81.27	100	7.73	8.60	1,221.6	21.79	28.8	4,091

WP, RYL and CPL of winter wheat crop are in million metric tons and ECL in million US dollars

The top three states Uttar Pradesh, Punjab, and Haryana contribute the highest winter wheat CPL 6 Mt (20 Mt) of the total winter wheat CPL 9 Mt (29 Mt) using M7 (AOT40) index. Ten states contribute the winter wheat ECL is 1,195 million US$ (4,048 million US$) of the total winter wheat ECL is 1,222 million US$ (4,091 million US$) using the M7 (AOT40) index. The comparison between M7 and AOT40 indices shows the winter wheat ECL is more than three times higher using AOT40 index than the winter wheat ECL using M7 index.

### Rabi rice losses and associated monetary loss

Table 4 shows the mean total rice (kharif plus rabi) production and rabi rice losses is estimated in the top ten states per year using the M7 and AOT40 indices for the study period. The production of rice in other states in India is low and it is shown as others (seven states) in Table 4. The state-wise order in Table 4 is as per the rabi rice production. The total mean rice production is 86 Mt per year (production of kharif rice is 74 Mt and rabi rice 12 Mt, i.e., 14 % of total rice production). The highest rice production is 15 and 11 Mt in West Bengal and in Uttar Pradesh, respectively. Similarly, the highest rabi rice production is 4.2 Mt in West Bengal and 3.9 Mt in Andhra Pradesh together account for about 70 % of the total rabi rice production. The highest rabi rice RYL is 0.24 Mt (0.62 Mt) in Andhra Pradesh due to high O_3_ concentration greater than 55 ppbv followed by in West Bengal is 0.21 Mt (0.55 Mt) of the total rabi rice RYL 0.63 Mt (1.7 Mt) per year using the M7 (AOT40) index. The total rabi rice RYL is 0.63 Mt (1.7 Mt) accounting for 5 % (14 %) losses using the M7 (AOT40) index per year of the total rabi rice production.

**Table 4:** Mean total rice (kharif plus rabi) production (RP) and rabi rice (RR) losses (relative yield loss (RYL), crop production loss (CPL), and economic cost loss (ECL)) per year in top ten states in India using M7 and AOT40 indices for the period 2002–2007

State	Rice production	RP (%)	Rabi rice production	RR (%)	Rabi rice losses					
					M7			AOT40		
					RYL	CPL	ECL	RYL	CPL	ECL
West Bengal	14.89	17.4	4.22	36.1	0.21	0.22	29.8	0.55	0.73	98.1
Andhra Pradesh	10.06	11.8	3.88	33.3	0.24	0.25	33.8	0.62	0.74	99
Karnataka	4.05	4.7	1.12	9.8	0.07	0.07	9.7	0.18	0.21	28.5
Orissa	6.11	7.1	0.61	5.3	0.02	0.03	3.4	0.08	0.09	12.6
Assam	3.65	4.3	0.59	5.2	0.02	0.03	3.3	0.08	0.09	12.5
Tamil Nadu	4.52	5.3	0.49	4.3	0.01	0.01	1.6	0.04	0.06	8.4
Bihar	4.2	4.9	0.17	1.7	0.02	0.01	0.1	0.02	0.03	3.5
Kerala	0.64	0.1	0.09	0.1	0.01	0.01	0.1	0.01	0.01	1.5
Maharashtra	2.51	2.9	0.07	0.1	0.01	0.01	0.1	0.01	0.01	1.3
Uttar Pradesh	11.2	13.1	0.01	0.1	0.01	0.01	0.1	0.01	0.01	0.1
Others (7 states)	23.84	27.9	0.46	4	0.03	0.03	4.2	0.07	0.08	10.9
Total	85.67	100	11.71	100	0.63	0.64	86.1	1.66	2.06	276.4

RP, rabi rice, RYL, and CPL are in million metric tons and ECL in million US dollars

*The highest rabi rice CPL is 0.3 Mt* (*0.74 Mt*) *in Andhra Pradesh followed by 0.2 Mt* (*0.73 Mt*) *in West Bengal of the total CPL 0.64 Mt* (*2.06 Mt*) *using the M7* (*AOT40*) *index*, *and the associated ECL is 34 million US*$ (*99 million US*$) *and 30 million US*$ (*98 million US*$) *of the total ECL is 86 million US*$ (*276 million US*$) *annually*. The comparison between M7 and AOT40 indices shows the rabi rice ECL is more than three times higher using the AOT40 index than the rabi rice ECL using the M7 index.

## Discussion

In this study, we estimate winter wheat and rabi rice crop yield losses using the M7 and AOT40 indices in India. The results obtained in this study are compared with previous studies carried out in India and on global scale reveals that our estimate of winter wheat and rabi rice losses due to O_3_-induced exposure are in good agreement. The average estimated winter wheat and rabi rice RYL is 9 (21 %) and 5 % (14 %), respectively, using the M7 (AOT40) index. The higher winter wheat RYL is 10 % (28 %) over the Indo-Gangetic plain using the M7 (AOT40) index in this study is comparable with the winter wheat RYL reported using the AOT40 index from measured O_3_ by Ghude et al. (2008). Ghude et al. (2008) and Deb Roy et al. (2009) reported that winter wheat RYL is 23 % over Indo-Gangetic plain in north India. The winter wheat and rabi rice losses estimated in this study is well agree with the winter wheat and summer crop yield losses 1.1–15.6 % is estimated from the O_3_ concentration using the AOT40 index in earlier studies in India (Mittal et al. 2007; Engardt 2008; Debaje et al. 2010; Elampari et al. 2013). Winter wheat and rabi rice losses estimated in this study is comparable to the grain weights of winter wheat (rice) is decreases from 18.6 to 37.2 % (10.1–17.3 %) with increase of AOT40 values from 23 to 62 ppm h (29–83 ppm h) in OTC study in China reported by Wang et al. (2012). Recently, Tang et al. (2013) projected using chemical transport model that the RYL of wheat to increase 13.6–30 % for India and 14.5–24.3 % in China based on AOT40 index in 2020. Our estimate of winter wheat and rabi rice losses is in the range of the grain losses projected due to increase O_3_ concentration using M7 and M12 indices to increase to 2–16 % for wheat and rice in 2020 in China, Japan, and South Korea from 1 to 9 % in 1990 by Wang and Mauzerall (2004). Further, Zhu et al. (2011) reported that the response of RYL of winter wheat to O_3_ concentration is similar in China, Europe, and India in an OTC study indicates that the increasing O_3_ is the rising threat to wheat production.

*The estimated rabi rice RYL is 5* % (*14* %) *using the M7* (*AOT40*) *index is in agreement with the losses in rice yield is 10*–*15* % *at 40*–*70 ppbv O*_*3*_ *concentration in an OTC study in Varanasi*, *India reported by* Rai et al. (2010). Our estimate for rabi rice losses in this study is similar to the RYL for rice is 6 % during summer season using AOT40 index in north India (Ghude et al. 2008). Similarly, Van et al. (2009) reported that the rice grain yield loss is 10 % at 32 ppbv and 17 % at 62 ppbv O_3_ concentration treatment in fumigation study at a periurban area of Vietnam is agree well with the our estimate of rabi rice in India.

The winter wheat and rabi rice RYL is 5–11 (6–30 %) and 3–6 % (12–16 %), respectively, based on M7 (AOT40) index in this study. The global studies performed by Van Dingenen et al. (2009) reported that winter wheat (rice) RYL is 13 and 28 % (6 and 8 %) using the M7 and AOT40 indices, respectively, over the India is well agree with the estimated winter wheat (rice) RYL in this study. Avnery et al. (2011a) estimate winter wheat RYL is 8 % (27 %) using the M7 (AOT40) index over the India is in good agreement with the estimate of winter wheat RYL in this study.

Table 5 shows the summary of winter wheat CPL and ECL estimated in this study is compared with studies available for India on global scale. The winter wheat CPL is 9 and 29 Mt (associated ECL is 1,222 and 4,091 million US$, mean ECL 2,657 million US$) using M7 and AOT40 indices, respectively, annually in this study are in good agreement with earlier estimate for winter wheat CPL is 12 and 29 Mt (associated ECL is 1,711 and 4,310 million US$, mean ECL 3,011 million US$ for the year 2000) for India reported by Van Dingenen et al. (2009). Similarly, winter wheat CPL is 8 and 25 Mt (associated ECL is 1,212 and 3,788 million US$, mean ECL 2,500 million US$ for the year 2000) using M7 and AOT40 indices, respectively, over India reported by Avnery et al. (2011a) is agreed well with the winter wheat CPL and ECL is estimated in this study.

**Table 5:** Summary and comparison of estimated winter wheat crop production loss (CPL) and associated economic cost loss (ECL) per year in India using M7 and AOT40 indices for the period 2002–2007

CPL			ECL			Reference
M7	AOT40	Mean	M7	AOT40	Mean	
8.6	28.8	18.7	1,222	4,091	2,657	Present study
11.6	29.1	20.4	1,711	4,310	3,011	Van Dingenen et al. (2009)
8	25	16.5	1,212	3,788	2,500	Avnery et al. (2011a, b)

Winter wheat CPL is in million metric tons, and ECL in million US dollars

We estimate rabi rice CPL is 0.64 Mt (2 Mt) and associated ECL is 86 million US$ (276 million US$) using the M7 (AOT40) index per annum for the study period. The estimated rabi rice losses in this study is not compared with the total rice (kharif plus rabi rice) losses (8–11 Mt) estimated using the M7 and AOT40 indices over India by Van Dingenen et al. (2009), as we estimate only rabi rice losses. We expect high critical limit 12.8 ppm h is not exceeding during the rainy season in India. Available O_3_ studies in India shows the measured seasonal diurnal variations of maximum O_3_ concentration is less than 30 ppbv during the monsoon season (June–September; kharif rice season) over the Indian region (Satsangi et al. 2004; Ahammed et al. 2006; Beig et al. 2007; Reddy et al. 2008; Debaje and Kakade 2009; Purkait et al. 2009; Debaje et al. 2010; Kumar et al. 2010; Singla et al. 2011; Mahapatra et al. 2012; David and Nair 2011; Nishanth et al. 2012; Elampari et al. 2013).

As Asian (including Indian) cultivars (wheat and rice) are more sensitive to O_3_ than the western CR functions, that highlight crop yield losses are underestimated in Asia (Emberson et al. 2009). The winter wheat RYL increases to 20 % with the increase of mean O_3_ concentration by 25 % in the ambient environment of China reported by Zhu et al. (2011). The crop yield losses are predicted to escalate in future due to climate change and increase of O_3_ concentration in many areas (IPCC 2001; Oltmans et al. 2006; The Royal Society 2008; Cape 2008; Wilkinson et al. 2012; Lobell and Gourdji 2012). Indian population is growing at the rate of 1.3 % per year (2012). The current India’s population is 1.2 billion and projected to increase to 1.6 billion (40 % more than it is now) in 2050 and then decline slowly to 1.5 billion in 2100 (UN 2013). To meet the food demand, the production of food grains in India needs to be increased at the rate of 6 Mt annually (ICAR 2011). The world will need 70 to 100 % more food to feed 9 billion people in 2050 than in 2006 (WDR 2008; Godfray et al. 2010; Tilman et al. 2011; WRI 2013). The projected demand for winter wheat and rabi rice (14 % of the total rice production) in India are increase to 95 and 22 Mt, respectively, in 2030 from 64 and 11 Mt in 2000 (ICAR 2011), while it is 138 and 34 Mt in 2050. The winter wheat (rabi rice) RYL is 31.1 % (11.3 % mean of M7 and AOT40 indices) in 2030 (Van Dingenen et al. 2009), and it is 41.8 % (15.6 %) in 2050 due to increase of O_3_ concentration under an optimistic scenario of future O_3_ precursor emissions. The winter wheat (rabi rice) CPL is 51 Mt (3.4 Mt) in 2030, and it is 99 Mt (6.6 Mt) in 2050, the associated winter wheat (rabi rice) ECL is 7.2 billion US$_2002–2007_ (0.4 billion US$_2002–2007_) in 2030, and it is 14 billion US$_2002–2007_ (0.9 billion US$_2002–2007_) in 2050 due to O_3_-induced yield losses. The projected winter wheat CPL is 51 Mt in 2030 in this study is comparable with estimated winter wheat CPL 56 Mt highest in India in 2030 reported by Avnery et al. (2011b). The total monetary loss occur is 2.8 billion US$_2002–2007_ per year for 2002–2007 due to winter wheat and rabi rice losses, while the losses 7.6 billion US$_2002–2007_ in 2030 and 14.9 billion US$_2002–2007_ in 2050. The climate change will reduce highest agriculture crop yield by 18 % in India in 2050 expected to further increase the food security problems (Wheeler and Braun 2010; WDR 2010). To feed a growing population, India will need to increase crop yield with sustainable intensification of agriculture. The winter wheat and rabi rice losses is estimated in this study should be view cautiously because of limited O_3_ data over the Indian region. Our results suggest that future winter wheat and rabi rice yield losses due to O_3_ damage will be large, if no mitigation efforts for curbing the O_3_ precursor emissions (particularly NO_x_) are effectively implemented in India.

## Conclusions

In this study, we estimated India’s risk to two major crops (winter wheat and rabi rice) of O_3_ pollution using measured O_3_ concentration and two indices (M7 and AOT40) of O_3_ exposure, field-based CR relationships. The estimated winter wheat and rabi rice RYL was 5–11 (6–30 %) and 3–6 % (12–16 %) of the total production of winter wheat 81 Mt and rabi rice 12 Mt, respectively, according to M7 (AOT40) index per annum for the period 2002–2007. *The average winter wheat and rabi rice RYL was 9* (*21* %) *and 5* % (*14* %), *respectively*, *estimated using the M7* (*AOT40*) *index. The estimated winter wheat and rabi rice CPL was 9* (*29 Mt*) *and 0.64 Mt* (*2 Mt*) *and associated ECL was 1,222* (*4,091 million US*$) *and 86* (*276 million US*$), *respectively, using M7* (*AOT40*) *index annually*. The wheat and rice are the main staple crops of the Indian people posing at high risk due to increasing O_3_ pollution. The M7 and AOT40 indices values are suggested that agricultural crops production in India is potentially at high risk from increasing O_3_ concentration indicated that the rise in anthropogenic pollution in the urban and rural areas.
